# Amyloid‐β Dysregulates Oligodendroglial Lineage Cell Dynamics and Myelination via PKC in the Zebrafish Spinal Cord

**DOI:** 10.1002/glia.70015

**Published:** 2025-03-14

**Authors:** Uxue Balantzategi, Adhara Gaminde‐Blasco, Christina A. Kearns, Laura Bayón‐Cordero, María Victoria Sánchez‐Gómez, José Luis Zugaza, Bruce Appel, Elena Alberdi

**Affiliations:** ^1^ Department of Neurosciences University of the Basque Country (UPV/EHU) Leioa Spain; ^2^ Achucarro Basque Center for Neuroscience Leioa Spain; ^3^ Department of Pediatrics, Section of Developmental Biology University of Colorado‐Anschutz Medical Campus Aurora Colorado USA; ^4^ Department of Genetics, Physical Anthropology and Animal Physiology University of the Basque Country (UPV/EHU) Leioa Spain; ^5^ IKERBASQUE Basque Foundation for Science Bilbao Spain

**Keywords:** Alzheimer's disease, amyloid‐β, myelin, oligodendrocytes, PKC, spinal cord, zebrafish

## Abstract

Soluble forms of amyloid‐β (Aβ) peptide have been proposed as candidates to induce oligodendrocyte (OL) and myelin dysfunctions in the early stages of Alzheimer's disease (AD) pathology. Nevertheless, little is known about how Aβ affects OL differentiation and myelination in vivo, and the underlying molecular mechanisms. In this study, we explored the effects of a brain intraventricular injection of Aβ on OLs and myelin in the developing spinal cord of zebrafish larvae. Using quantitative fluorescent in situ RNA hybridization assays, we demonstrated that Aβ altered *myrf* and *mbp* mRNA levels and the regional distribution of *mbp* during larval development, suggesting an early differentiation of OLs. Through live imaging of *Tg*(*myrf:mScarlet*) and *Tg*(*mbpa:tagRFP*) zebrafish lines, both crossed with *Tg*(*olig2:EGFP*), we found that Aβ increased the number of *myrf* ^+^ and *mbp* ^+^ OLs in the dorsal spinal cord at 72 hpf and 5 dpf, respectively, without affecting total cell numbers. Furthermore, Aβ also increased the number of Sox10^+^cells, myelin sheaths per OL, and the number of myelinated axons in the dorsal spinal cord at 8 dpf compared to vehicle‐injected control animals. Interestingly, the treatment of Aβ‐injected zebrafish with the pan‐PKC inhibitor Gö6983 restored the aforementioned alterations in OLs and myelin to control levels. Altogether, not only do we demonstrate that Aβ induces a precocious oligodendroglial differentiation leading to dysregulated myelination, but we also identified PKC as a key player in Aβ‐induced pathology.

## Main Points

- Amyloid β peptide induces early oligodendrocyte differentiation and dorsal myelination in the zebrafish larvae spinal cord.
- Pan‐PKC inhibitor Gö6983 restores the aforementioned alterations in oligodendrocytes and myelin.

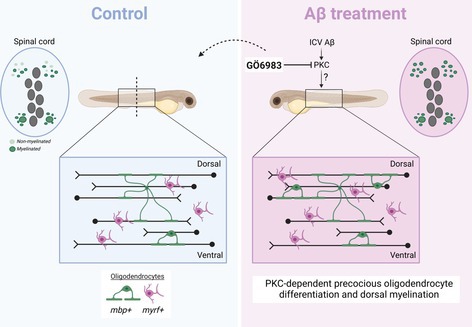

## 1 Introduction

Oligodendrocytes (OLs) are specialized glial cells responsible for forming myelin sheaths around axons in the central nervous system (CNS). This myelination is essential for the rapid propagation of electrical signals and overall neuronal connectivity (Baumann and Pham‐Dinh 2001). Dysfunction of OLs and myelin is among the early pathological events observed in Alzheimer's disease (AD), a chronic neurodegenerative condition primarily characterized by cognitive decline and memory loss (Desai et al. 2009; Roher et al. 2002). Although there is strong evidence connecting OL impairment with the onset of neurodegeneration in AD, the underlying causes of OL dysfunction in this disorder are poorly understood.

One of the major pathological hallmarks of AD is the accumulation of amyloid‐β (Aβ) soluble forms, which are thought to play a critical role in the disruption of OL differentiation and myelination processes (Horiuchi et al. 2012; Mitew et al. 2010). Notably, soluble forms of Aβ induced the expression of myelin basic protein (MBP), a key and essential myelin protein, and promoted OL proliferation and differentiation in vitro and in organotypic slices (Quintela‐López et al. 2019). These findings suggest that OL functions may be perturbed in AD. Nevertheless, only a few studies have investigated the role of OLs in AD and proposed Aβ as a candidate for promoting white matter dysfunction (Dean et al. 2017; Selkoe and Hardy 2016), but the mechanisms remain unclear.

Protein kinase C (PKC) is a family of kinases that play essential roles in various physiological processes to maintain cell homeostasis, including cell cycle regulation (Nelson and Alkon 2009), differentiation (Cavaliere et al. 2013; Damato et al. 2021), and apoptosis. Interestingly, several studies have shown that PKC activation can influence OL differentiation, inducing the expression of myelin‐related genes and promoting myelination (Asotra and MacKlin 1993; Swire et al. 2019). Moreover, PKC is involved in the pathogenesis of a wide range of disorders, including cancer (Garg et al. 2014) and neurological disorders such as AD (Alfonso et al. 2016; Newton 2010). Furthermore, previous investigations have reported increased PKC activity in response to Aβ in both astrocytes (Abramov and Duchen 2005; Wyssenbach et al. 2016) and neurons (Manterola et al. 2013; Ortiz‐Sanz et al. 2022). However, Aβ‐induced PKC activation has not been explored for OLs.

Zebrafish ( *Danio rerio* ) are increasingly recognized as a valuable model organism for studying OLs and myelin in vivo, since their embryos and larvae are transparent, allowing for real‐time imaging of myelination and OL dynamics (Czopka 2016; Kimmel et al. 1995). In addition, zebrafish have a high degree of genetic and physiological homology with humans, particularly in the CNS, making them a relevant model for human neurological diseases (Howe et al. 2013; Lieschke and Currie 2007). In fact, myelin genes exhibit a 70%–90% homology between human and zebrafish (Howe et al. 2013). One key feature of zebrafish is their rapid early development. Unlike mammals, where myelination occurs primarily during postnatal stages, zebrafish exhibit early initiation of myelination, providing a condensed timeline for studying this crucial process. In zebrafish, oligodendrocyte precursor cells (OPCs) emerge as early as 48 h postfertilization (hpf) and subsequently differentiate into mature OLs capable of myelination. By 60 hpf, OLs begin to express key myelin‐related genes such as myelin basic protein (*mbp*) or myelin protein zero (*mpz/p0*) (Brösamle and Halpern 2002; Buckley et al. 2010; Park et al. 2002) This rapid development makes zebrafish a valuable model for studying oligodendrogenesis and myelination.

In this study, we investigated whether intracerebroventricular (ICV) injection of Aβ into zebrafish larvae affects OL differentiation, dynamics, and myelination in vivo, and whether PKC is involved in the underlying signaling pathway. Our results indicated that Aβ alters *myrf* and *mbp* mRNA levels and the regional distribution of *mbp* in the developing spinal cord of zebrafish larvae. Live imaging revealed that Aβ increased the number of *myrf* ^+^ and *mbp* ^+^ OLs in the dorsal spinal cord without affecting the total cell number, suggesting premature differentiation and migration of OLs. Additionally, Aβ increased the number of OLs, myelin sheaths per OL, and the number of myelinated axons in the dorsal spinal cord without affecting the sheath length. Treatment with the pan‐PKC inhibitor Gö6983 reversed the effects of Aβ to physiological levels, confirming that Aβ dysregulated OLs and myelin through PKC function. These results provide new insights into the in vivo effects of Aβ on OLs and highlight PKC as a promising therapeutic target in AD.

## 2 Materials and Methods

### 2.1 Zebrafish

Nontransgenic embryos were obtained through crosses of male and female zebrafish from the AB strain. Embryos were raised at 28.5°C in E3 media (5 mM NaCl, 0.17 mM KCl, 0.33 mM CaCl, 0.33 mM MgSO_4_ (pH 7.4), supplemented with sodium bicarbonate). Larvae were staged according to hours or days postfertilization (hpf/dpf) and selected based on criteria ensuring good health and normal developmental patterns. For cell count experiments, the previously established zebrafish lines *Tg*(*olig2:EGFP*)^*vu12*^, *Tg*(*myrf:mScarlet*)^*co66*^, and *Tg*(*mbpa:tagRFPT*)^*co25*^ were used.

### 2.2 Preparation of Soluble Amyloid‐β

Soluble amyloid‐β (Aβ) was prepared as previously described (Dahlgren et al. 2002). Briefly, Aβ_1–42_ (Bachem, Germany) was initially dissolved to a concentration of 1 mM in hexafluoroisopropanol (Sigma‐Aldrich), which was next totally removed under vacuum using a speed‐vac system. The resulting peptide film was stored desiccated at −80°C. For the aggregation protocol, the peptide was resuspended in dry dimethylsulfoxide (DMSO; Sigma‐Aldrich) to achieve a concentration of 5 mM, and Hams F‐12 (PromoCell) was then added to adjust the peptide to a final concentration of 100 μM. Vehicle was composed of 1.8% DMSO in Hams F‐12.

Oligomer formation was induced by incubating the peptide solution for 24 h at 4°C, and a mix of monomers, trimers, and tetramers of Αβ peptide was confirmed by polyacrylamide gel electrophoresis (SDS‐PAGE), followed by Coomassie Brilliant Blue R‐250 (Bio‐Rad) staining. The characterization of this Aβ preparation by transmission electron microscopy showed mainly Aβ oligomers and very few protofibrils (Alberdi et al. 2018).

Fluorescently labeled Aβ_1‐42_ was prepared as previously described (Jungbauer et al. 2009). After 24 h incubation at 4°C, labeling was performed using the Alexa Fluor 488 Microscale Protein Labeling Kit (#A30006, Invitrogen) according to the manufacturer's instructions. Briefly, 50 μL of 100 μM Aβ solution was adjusted to pH 9 with 5 μL of 1 M NaHCO_3_, followed by the addition of 4 μL of the H_2_O solubilized reactive dye. Incubation at room temperature was performed for 15 min, followed by the immediate addition of 55 μL of the labeling reaction mixture onto a spin column packed with a 425 μL slurry of Biogel P‐6 resin for the removal of unincorporated dye. The resulting eluent (pH 7.4) was stored for up to 2 days at 4°C or used immediately for injections.

### 2.3 Intracerebroventricular Amyloid‐β Injection and Drug Treatment in Zebrafish Larvae

At 24 hpf, all zebrafish embryos had their chorions removed for the brain ventricle injection procedure following previously established protocols (Gutzman and Sive 2009; Nery et al. 2014). Embryos were anesthetized with Tricaine (Sigma‐Aldrich) and immobilized in wells on 2%‐agar coated dishes under the stereomicroscope so that the brain ventricle was exposed. An injection needle was carefully positioned on the roof plate of the hindbrain, and 5–10 nL of a fresh injection buffer containing 10% either Aβ (10 μM) or vehicle, and 10% Phenol Red in 0.4 M KCl in nuclease‐free water was microinjected. Subsequently, each treatment group was separated into clean plates, and the zebrafish were returned to the incubator and allowed to grow until the days of the experiments. For PKC inhibition, zebrafish larvae were treated with vehicle (0.005% DMSO) or Gö6983 (500 nM, Tocris) by bath immersion at 48 hpf.

### 2.4 RNA In Situ Hybridization

To identify potential Aβ‐induced alterations in OL differentiation‐ and myelination‐related gene expression in vivo, we conducted fluorescent RNA in situ hybridization (FISH) assays in the spinal cords of zebrafish larvae injected with either vehicle or Aβ (10 μM). FISH was performed using the RNAScope Multiplex Fluorescent V2 Assay Kit (Advanced Cell Diagnostics). Zebrafish larvae at 48 hpf, 72 hpf, and 5 dpf were fixed in 4% paraformaldehyde (PFA) in PBS, gently rocking overnight at 4°C. Subsequently, samples were embedded in 1.5% agar with 30% sucrose, followed by immersion in 30% sucrose overnight. The blocks were frozen on dry ice, and 15 μm‐thick transverse sections were obtained using a cryostat microtome and collected on polarized slides.

FISH was performed according to the manufacturer's instructions, with the following modification: slides were covered with Parafilm for all 40°C incubations to maintain moisture and disperse reagents across the sections. The zebrafish *mbpa*, *myrf*, and *sox10* transcript probes were designed and synthesized by the manufacturer and used at 1:50 dilutions, except for *mbpa*, which was used undiluted. The transcripts were fluorescently labeled by TSA‐based Opal fluorophores Opal520 (1:1500), Opal570 (1:500), and Opal650 (1:1500) using the Opal 7 Kit (PerkinElmer). Sections were incubated with DAPI for 30 s and mounted in Vectashield (Vector Laboratories, H‐1000‐10).

Images were acquired on a Zeiss Cell Observer 2D 25 Spinning Disk confocal system (Carl Zeiss Microscopy) with a 40× oil‐immersion objective. 15 z‐stack tiles (z‐step = 0.5 μm) of 5 sections of the spinal cord were acquired from each animal. The area occupied by each probe was quantified using ImageJ/Fiji. The dorsal and ventral regions were defined according to DAPI staining patterns, as previously described (Cucun et al. 2024).

### 2.5 In Vivo Myelin Sheath Visualization

To visualize myelin sheaths in vivo, the plasmid *pEXPRS‐mbpa:EGFP‐CAAX.tol2* was transiently expressed by microinjection with synthetic mRNA encoding Tol2 transposase into 1‐cell stage nontransgenic zebrafish embryos. Next, zebrafish larvae were ICV injected with either vehicle or Aβ (10 μM) at 24 hpf, and treated by bath immersion with or without Gö6983 (500 nM, Tocris) at 72 hpf. At 5 dpf, zebrafish larvae were anesthetized with Tricaine (Sigma‐Aldrich) and embedded laterally in 1% low‐melt agarose containing 0.4% Tricaine for immobilization on a glass bottom dish.

Images were acquired using a Zeiss Cell Observer 2D 25 Spinning Disk confocal system (Carl Zeiss Microscopy) with a 40× water‐immersion objective. Images were collected from 1 to 3 fields of view from each animal in 40 z‐stack tiles (z‐step = 0.28 μm). Subsequent analysis of sheath length and total number of sheaths per OLs was performed using Imaris software v9.9.1 (Oxford Instruments) in a blind mode.

### 2.6 Electron Microscopy (EM)

At 8 dpf, vehicle‐ or Aβ‐injected (10 μM) zebrafish larvae were anesthetized with Tricaine and fixed in a solution consisting of 2% glutaraldehyde and 4% PFA in 0.1 M sodium cacodylate (pH 7.4) for at least 5 days at 4°C. The tissue was then sagittally sectioned using a Leica VT 1200S vibrating blade microtome (Leica) to obtain 200 μm‐thick sections. These tissue sections were postfixed in 2% OsO4, dehydrated in ethanol and propylene oxide, and embedded in EPON (Serva) for 24 h at 60°C. Ultrathin sections (50 nm thickness) were obtained using a Leica Ultracut S ultramicrotome (Leica) and contrasted for 30 min with 4% uranyl acetate and 6 min with lead citrate.

Electron micrographs were captured using a Zeiss EM900 electron microscope (Carl Zeiss Microscopy). The number and diameter of dorsal myelinated axons were quantified using ImageJ/Fiji in a blind mode.

### 2.7 Immunohistochemistry

For Sox10 cell quantification by immunohistochemistry, 8 dpf *Tg*(*olig2:EGFP*)^*vu12*^ embryos were fixed in 4% PFA, embedded, frozen, and sectioned using a cryostat microtome as previously described (Park and Appel 2003). Sections were incubated with the primary antibody rabbit anti‐Sox10 (1:500) (Park et al. 2005), followed by fluorescent detection of antibody labeling using Alexa Fluor 568‐conjugated goat antirabbit conjugate (Invitrogen, 1:200).

Images were acquired using a Zeiss Cell Observer 2D 25 Spinning Disk confocal microscope (Carl Zeiss Microscopy) with a 40× oil‐immersion objective. 10 serial sections of the spinal cord were analyzed per embryo, and quantifications were performed in a blinded manner.

### 2.8 Statistical Analysis

All data are presented as mean ± SEM (standard error of the mean), with sample size indicated in the figures by dots. Statistical analyses were performed using absolute values. GraphPad Prism 8.2.1 software was utilized, applying unpaired two‐tailed Student's t‐test for comparing two experimental groups, and one‐way or two‐way analysis of variance (ANOVA) followed by Tukey's and Sidak's *post hoc* tests for multiple comparisons. Results from independent animals were considered biological replicates (*n* ≥ 5). Statistical significance was represented as *p* < 0.05 (*), *p* < 0.01 (**), *p* < 0.001 (***), and *p* < 0.0001 (****).

## 3 Results

Emerging evidence suggests that alterations in oligodendrogenesis and disruptions in myelin integrity are key early events in AD pathology (Ferreira et al. 2020; Wu et al. 2017). Previous studies have shown that Aβ peptide promotes OL differentiation and maturation in primary culture rat OLs and organotypic cerebellar slices (Quintela‐López et al. 2019). Therefore, our main goal was to investigate the potential effects of ICV injection of Aβ on OL differentiation and myelination in vivo in zebrafish larvae.

**FIGURE 1:**
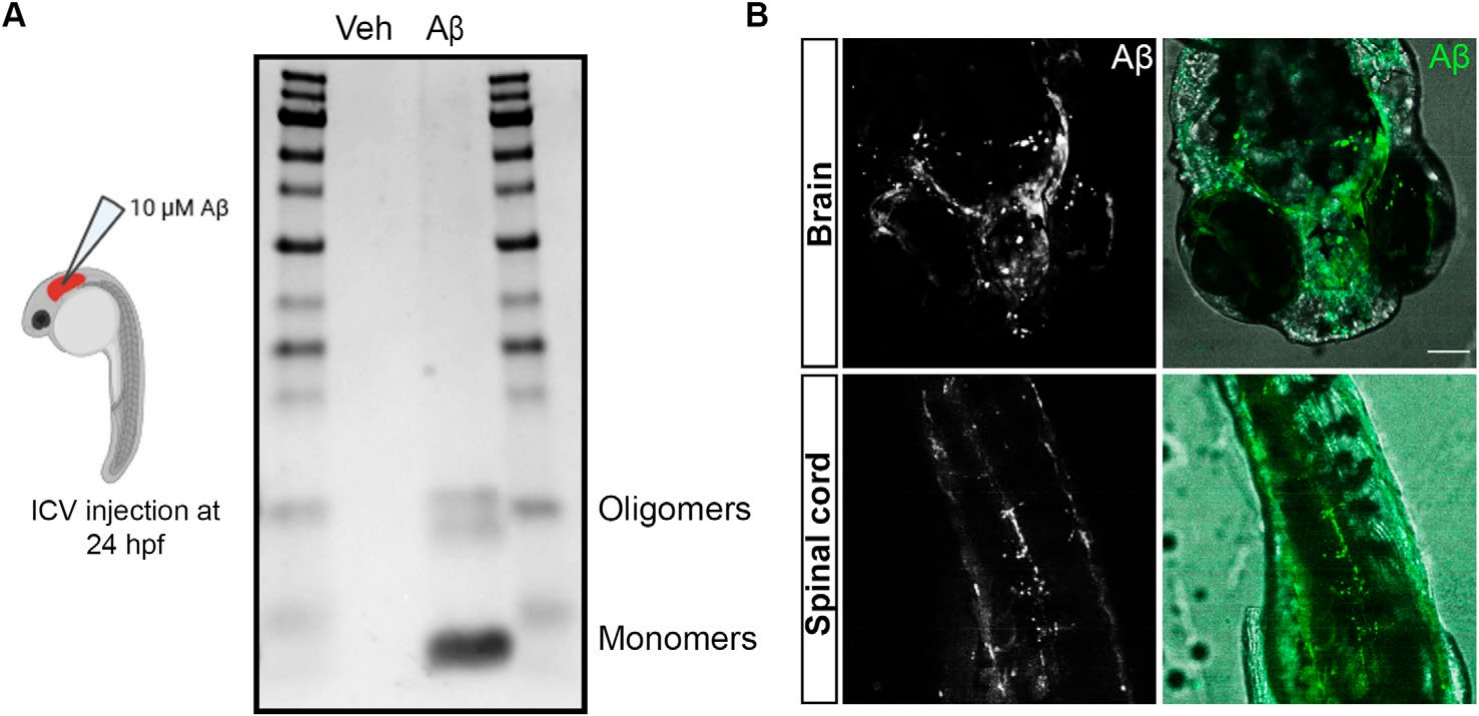
Intracerebroventricular Aβ injection into zebrafish larvae. (A) Schematic representation of the experimental approach, and the detection of Aβ species (monomers and oligomers) in the injection mixtures of vehicle and Aβ using Coomassie blue staining. (B) Representative images of fluorescent‐labeled Aβ diffusion into the brain and spinal cord of 24 hpf zebrafish larvae following ICV injection. Scale bar = 50 μm.

First, Aβ molecular species were confirmed using in‐gel Coomassie blue staining (Figure 1A). In addition, injection efficiency was assessed by injecting fluorescently labeled dextran (Figure S1) and Aβ (Figure 1B) into the hindbrain ventricle of 24 hpf zebrafish larvae. We observed a widespread distribution of the injection mixture throughout the brain and spinal cord of the larvae. Therefore, ICV injections were considered adequate to proceed with the proposed experimental procedures.

### 3.1 Aβ Induces Early Oligodendrocyte Differentiation and Maturation in Zebrafish

**FIGURE 2:**
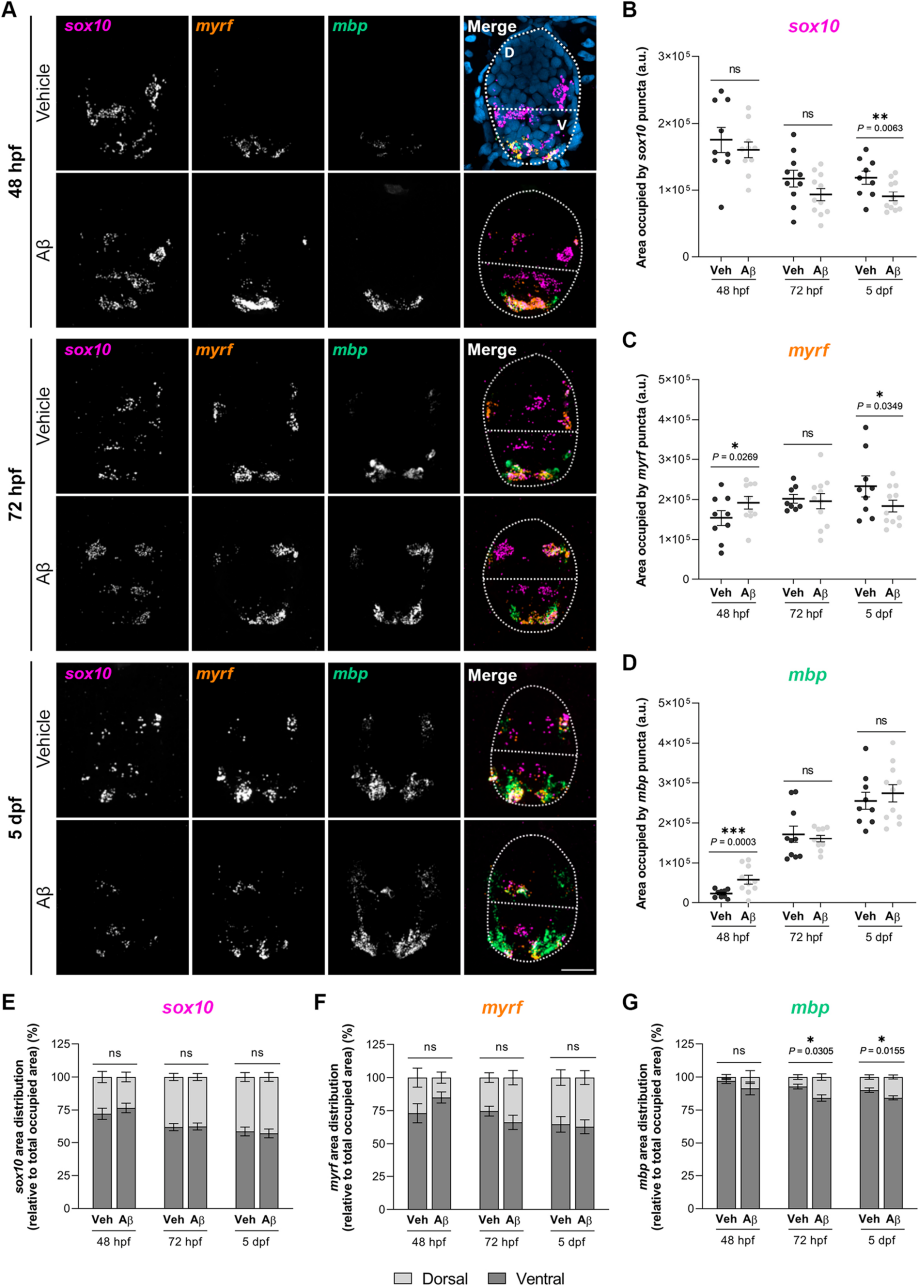
Aβ‐injected zebrafish larvae exhibit alterations in oligodendroglial lineage mRNA levels and regional distribution in the developing spinal cord. Zebrafish larvae were injected with Aβ or its vehicle at 24 hpf, and FISH assays were performed for *sox10*, *myrf* and *mbp* at 48 hpf, 72 hpf and 5 dpf. (A) Representative confocal images of spinal cord cross‐sections depicting mRNA expression fluorescence. Dorsal and ventral regions were defined based on the DAPI (blue) pattern, and are delineated by the dotted white line. (B–D) RNAscope analysis representing the total area occupied by each mRNA in the developing spinal cord. (E–G) Quantification of the regional distribution of each mRNA in the ventral and dorsal regions, expressed relative to the total occupied area. Scale bar = 20 μm. Data are represented as means ± SEM, *p < 0.05, **p < 0.01, ***p < 0.001 compared to vehicle‐injected zebrafish larvae. Statistical significance was drawn by two‐tailed nested *t*‐test and two‐way ANOVA, followed by Sidak's *post hoc* test. *n* = 8–11 larvae per condition and time point.

To identify Aβ‐induced alterations in OL differentiation and maturation, we conducted fluorescent RNA in situ hybridization (FISH) assays following Aβ or vehicle ICV injections. The expression of three key genes associated with different developmental stages of the OL lineage was assessed: *sox10*, a canonical marker of the OL lineage; *myrf*, a transcriptional factor that regulates the expression of myelin‐related genes in differentiating OLs (Hornig et al. 2013); and *mbpa* (*mbp*), a zebrafish orthologue of murine and human *MBP*, expressed in myelinating mature OLs (Figure 2A).

The total area occupied by *sox10* decreased during the initial days of larval development, particularly in Aβ‐treated zebrafish, which exhibited reduced expression levels at 5 dpf compared to controls (1.18 ± 0.1 × 10^5^ a.u. in vehicle‐treated larvae vs. 0.9 ± 0.07 × 10^5^ a.u. in Aβ‐treated larvae) (Figure 2B). On the other hand, interestingly, *myrf* expression was significantly increased by ~25% at 48 hpf in the presence of Aβ (1.54 ± 0.19 × 10^5^ a.u. vehicle‐treated vs. 1.92 ± 0.16 × 10^5^ a.u. Aβ‐treated larvae). However, while *myrf* mRNA showed the expected increase in expression throughout development in control larvae, Aβ treatment induced a stable *myrf* expression over time, resulting in a ~21% reduction in expression at 5 dpf compared to vehicle‐injected larvae (2.33 ± 0.27 × 10^5^ a.u. vehicle vs. 1.84 ± 0.15 × 10^5^ a.u. Aβ) (Figure 2C). Consistent with the increased *myrf* levels at 48 hpf, there was a notable ~147% increase in *mbp* expression levels at the same time point in the presence of Aβ (2.33 ± 0.33 × 10^4^ a.u. vehicle vs. 5.77 ± 1.08 × 10^4^ a.u. Aβ). However, this early boost of myelin‐related mRNA was normalized by 72 hpf and remained at control levels throughout development (Figure 2D).

In zebrafish spinal cord development, the majority of OPCs originate from progenitor cells in the pMN domain located in the ventral spinal cord, and they subsequently migrate toward the dorsal zone, where they proliferate and differentiate into OLs (Lee et al. 2010). Consequently, both differentiation‐related gene expression and OPC migration are crucial events for proper OL differentiation and CNS development. Here, we measured the dorsoventral regional distribution of *sox10*‐, *myrf*‐, and *mbp*‐positive puncta during development. No changes were observed in the distribution of *sox10* (Figure 2E) or *myrf* (Figure 2F) mRNA following Aβ treatment. However, a significant shift in *mbp* expression toward the dorsal area was detected in Aβ‐injected larvae at both 72 hpf (7.2% ± 1.82% of the total area occupied by *mbp* was dorsal in vehicle‐injected larvae vs. 15.9% ± 2.45% in Aβ‐injected larvae) and 5 dpf (vehicle 9.95% ± 1.68% vs. Aβ 15.77% ± 1.56%) (Figure 2G).

Overall, these results suggest that Aβ may induce a precocious OL differentiation and maturation by altering the expression and regional distribution of differentiation‐ and myelination‐related mRNAs during larval development.

### 3.2 Aβ‐Induced Deregulation of Oligodendrocyte Differentiation and Maturation Timing Is PKC‐Dependent

Considering previous studies indicating increased PKC activity in response to Aβ in both astrocytes (Abramov and Duchen 2005; Wyssenbach et al. 2016) and neurons (Manterola et al. 2013; Ortiz‐Sanz et al. 2022), we next investigated the implication of PKC in Aβ‐induced effects in OLs. Increased phosphorylation levels of PKC were found in primary cultured OLs treated with Aβ for 3 or 24 h assessed via western blot analysis (141.3% ± 10.09% after 3 h of Aβ treatment and 144.5% ± 12.38% after 24 h of Aβ treatment, compared to 100% in control cells), indicating Aβ‐induced PKC activation in OLs (Figure S2).

**FIGURE 3:**
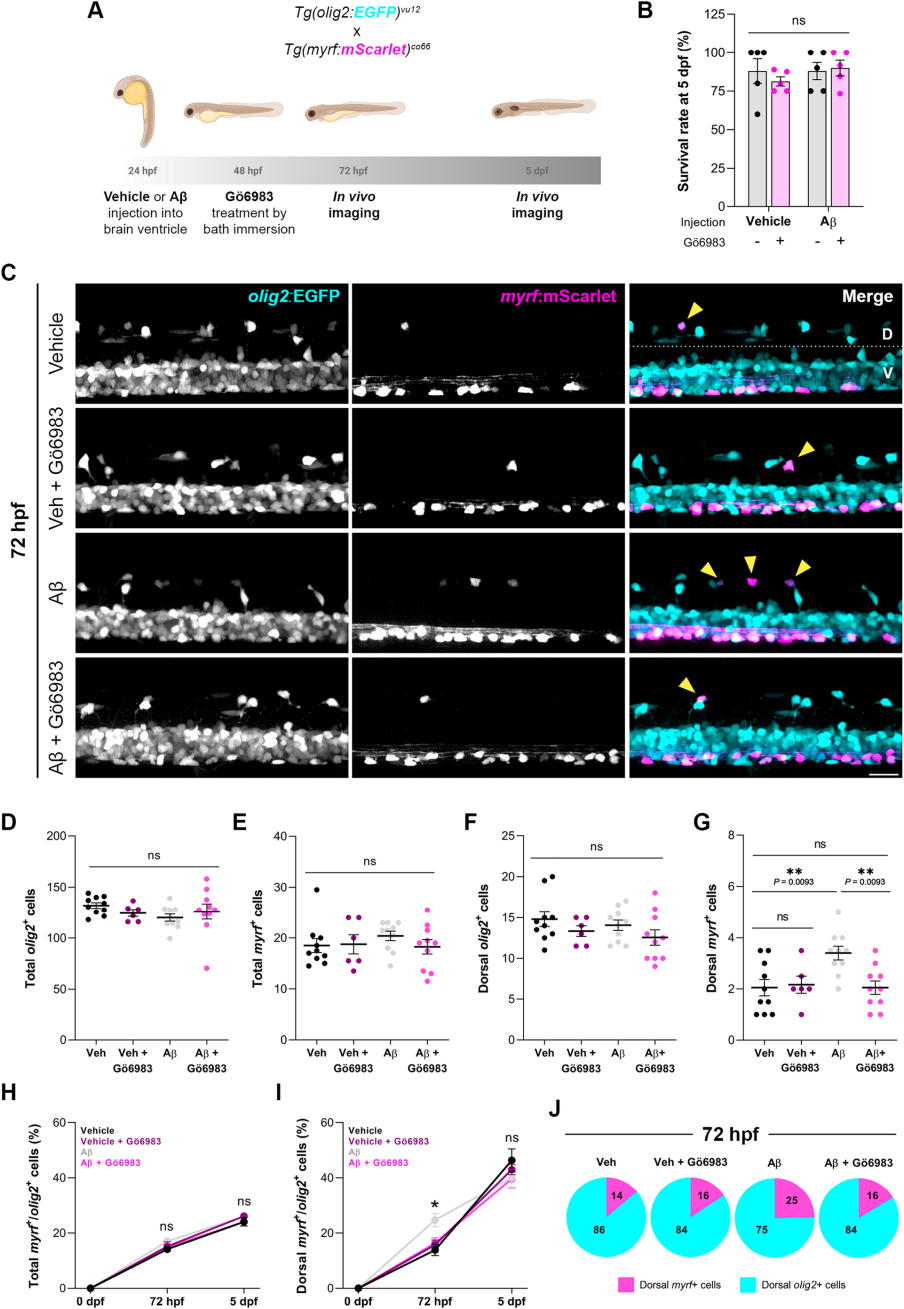
Aβ induces early oligodendrocyte differentiation through PKC, without affecting total cell numbers. (A) Transgenic zebrafish stably expressing *olig2*:EGFP and *myrf*:MScarlet were injected with Aβ or its vehicle at 24 hpf, and treated with Gö6983 (500 nM) at 48 hpf by bath immersion. Live imaging was performed at 72 hpf and 5 dpf. (B) Survival rate was measured at 5 dpf. (C) Representative lateral fluorescence images of the spinal cord in live transgenic larvae at 72 hpf. Yellow arrowheads indicate *olig2* ^+^ *myrf* ^+^ cells. Dorsal and ventral regions are delineated by the dotted white line. (D) Graphs comparing the number of total *olig2* ^+^ cells, (E) total *myrf* ^+^ cells, (F) dorsal *olig2* ^+^ cells, and (G) dorsal *myrf* ^+^ cells. (H) Graphs showing the progression of differentiating OLs (*myrf* ^+^/*olig2* ^+^ cells) in the total and (I) dorsal spinal cord. (J) Pie charts showing the ratio of differentiating dorsal OLs at 72 hpf for each condition. Scale bar = 20 μm. Data indicate means ± SEM, and dots represent individual larvae. *p < 0.05; Statistical significance was determined by two‐way ANOVA and mixed‐effects analysis followed by Tukey's *post hoc*. *n* ^72 hpf^ = 6–10 larvae per condition; *n* ^5 dpf^ = 7–9 larvae per condition.

To further investigate Aβ‐induced alterations in OL differentiation in greater detail, we conducted in vivo studies using stable transgenic zebrafish lines. Specifically, we crossed *Tg*(*olig2:EGFP*)^*vu12*^, a reporter of pMN lineage cells, with *Tg*(*myrf:mScarlet*)^*co66*^, a reporter of differentiating OLs. In addition to ICV injection of Aβ or vehicle at 24 hpf, larvae were also treated with or without the pan‐PKC inhibitor Gö6983 at 48 hpf (Figure 3A). The combination of Aβ and drugs did not show any noticeable significant toxicity for the larvae (Figure 3B).

At 72 hpf, no significant differences were observed in the total number of *olig2* ^+^ (Figure 3C,D) or *myrf* ^+^ cells (Figure 3E) across treatments. Nevertheless, when focusing on the dorsal spinal cord, while *olig2* ^+^ cell counts remained consistent across all treatment groups (Figure 3F), we detected a notable increase in dorsal *myrf* ^+^ cells in larvae injected with Aβ compared to those receiving vehicle injections. Interestingly, while treatment with Gö6983 alone did not alter dorsal *myrf* ^+^ cell numbers in vehicle‐treated larvae, it effectively attenuated the Aβ‐induced increase, restoring *myrf* ^+^ cell numbers to control levels (vehicle 2.05 ± 0.32 cells; Gö6983 2.17 ± 0.33 cells; Aβ 3.4 ± 0.27 cells; Aβ + Gö6983 2.05 ± 0.26 cells) (Figure 3G).

No significant changes were observed in differentiating *myrf* ^+^ OL numbers at 5 dpf (Figure S3). Therefore, the sequential study of OL differentiation in this transgenic line suggested that, while the total number of differentiating OLs (*myrf* ^+^/*olig2* ^+^ cells) remains unchanged (Figure 3H), the changes are localized to the dorsal region (Figure 3I). In the dorsal spinal cord, Aβ induces a transient increase in premature OL differentiation around 72 hpf, which returns to normal levels by 5 dpf. This effect was reversed by PKC inhibition (Figure 3I). Specifically, while only 14% of dorsal OLs were *myrf* ^+^ in vehicle‐treated larvae, this proportion increased to 25% following Aβ treatment and was restored to 16% when Aβ‐injected larvae were treated with Gö6983. Notably, Gö6983 alone had no significant effect (Figure 3J).

**FIGURE 4:**
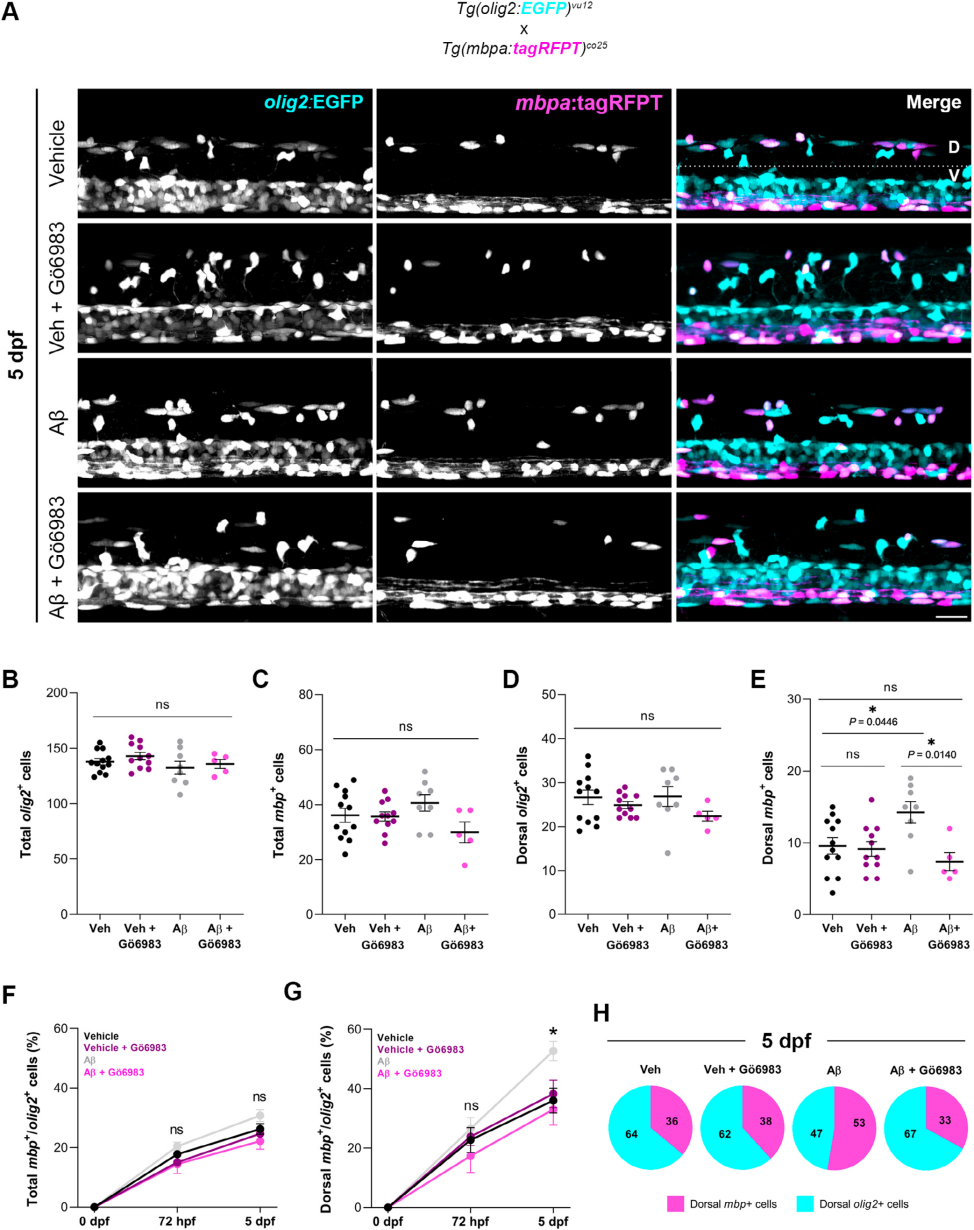
Aβ promotes oligodendrocyte maturation via PKC, without changing total cell numbers. (A) Representative lateral images of the spinal cord of live transgenic larvae stably expressing *olig2*:EGFP and *mbpa*:TagRFPT, at 5 dpf. Dorsal and ventral regions are delineated by the dotted white line. (B) Graphs comparing the quantity of total *olig2* ^+^ cells, (C) total *mbp* ^+^ cells, (D) dorsal *olig2* ^+^ cells, and (E) dorsal *mbp* ^+^ cells. (F) Graphical representation of the progression of maturing *mbp* ^+^ OLs (*mbp* ^+^/*olig2* ^+^ cells) throughout development in the total and (G) dorsal spinal cord. (H) Pie charts showing the percentages of dorsal mature OLs at 5 dpf. Scale bar = 20 μm. Data indicate means ± SEM, and dots represent individual larvae. *p < 0.05; Statistical significance was drawn by two‐way ANOVA and mixed‐effects analysis followed by Tukey's *post hoc*. *n* ^72 hpf^ = 6–12 larvae per condition; *n* ^5 dpf^ = 5–12 larvae per condition.

The above data suggest that a higher proportion of OPCs undergo differentiation into mature myelinating OLs in Aβ‐injected larvae. To directly test this possibility, we employed a similar approach as previously described, now crossing *Tg*(*olig2:EGFP*)^*vu12*^ with *Tg*(*mbpa:tagRFPT*)^*co25*^, a reporter line for mature myelinating OLs (Figure 4A). Since changes in *myrf* expression were observed at 72 hpf and OL maturation occurs later in time, it was expected that no significant changes in the number of mature myelinating OLs would be detected at 72 hpf (Figure S4). Thus, we focused our analysis on 5 dpf to examine mature myelinating OLs.

At this developmental stage, Aβ‐injected larvae exhibited an excess of *mbp* ^+^ cells in the dorsal spinal cord compared to vehicle‐injected larvae (Figure 4E), with no changes in *olig2* ^+^ (Figure 4B,D) or total *mbp* ^+^ cells (Figure 4C). As previously observed, Gö6983 treatment did not affect cell counts in vehicle‐injected larvae but inhibited the Aβ‐induced increase in *mbp* ^+^ cells (vehicle 9.58 ± 1.13 cells; Gö6983 9.18 ± 1.02 cells; Aβ 14.25 ± 1.5 cells; Aβ + Gö6983 7.4 ± 1.25 cells) (Figure 4E).

Sequential analysis of OL maturation using this transgenic line revealed that, while the total number of mature OLs (*mbp* ^+^/*olig2* ^+^ cells) remained unchanged (Figure 4F), Aβ significantly promoted OL maturation at 5 dpf in the dorsal spinal cord (Figure 4G), an effect that was effectively reversed by the PKC inhibitor Gö6983 (Figure 4G). Specifically, the proportion of *mbp* ^+^ OLs among dorsal OLs was 36% and 38% in vehicle‐injected control and Gö6983‐treated larvae, respectively, compared to 53% in Aβ‐treated larvae. This Aβ‐induced increase was reduced to 33% upon PKC inhibition (Figure 4H). No statistically significant changes were detected when analyzing the total spinal cord (Figure 4F).

Taken together, these data suggest that Aβ induces precocious OL differentiation, resulting in altered maturation. Furthermore, Gö698 effectively counteracts these changes, supporting the involvement of the PKC signaling pathway in Aβ‐induced dysregulation of OL dynamics.

### 3.3 Aβ Induces Myelin Excess in the Dorsal Spinal Cord Through PKC Activation

Next, we investigated whether Aβ‐induced OL differentiation and maturation could affect myelination in vivo. To assess myelin sheaths in individual OLs, we transiently expressed *mbpa*:EGFP‐CAAX by microinjection into 1‐cell stage zebrafish embryos. This genetic construct leads to the expression of membrane‐tethered EGFP in the myelin tracts of the larvae. Aβ or vehicle injections were administered, followed by Gö6983 treatments, and myelin sheath number per cell and sheath length were measured in the dorsal spinal cord at 5 dpf.

**FIGURE 5:**
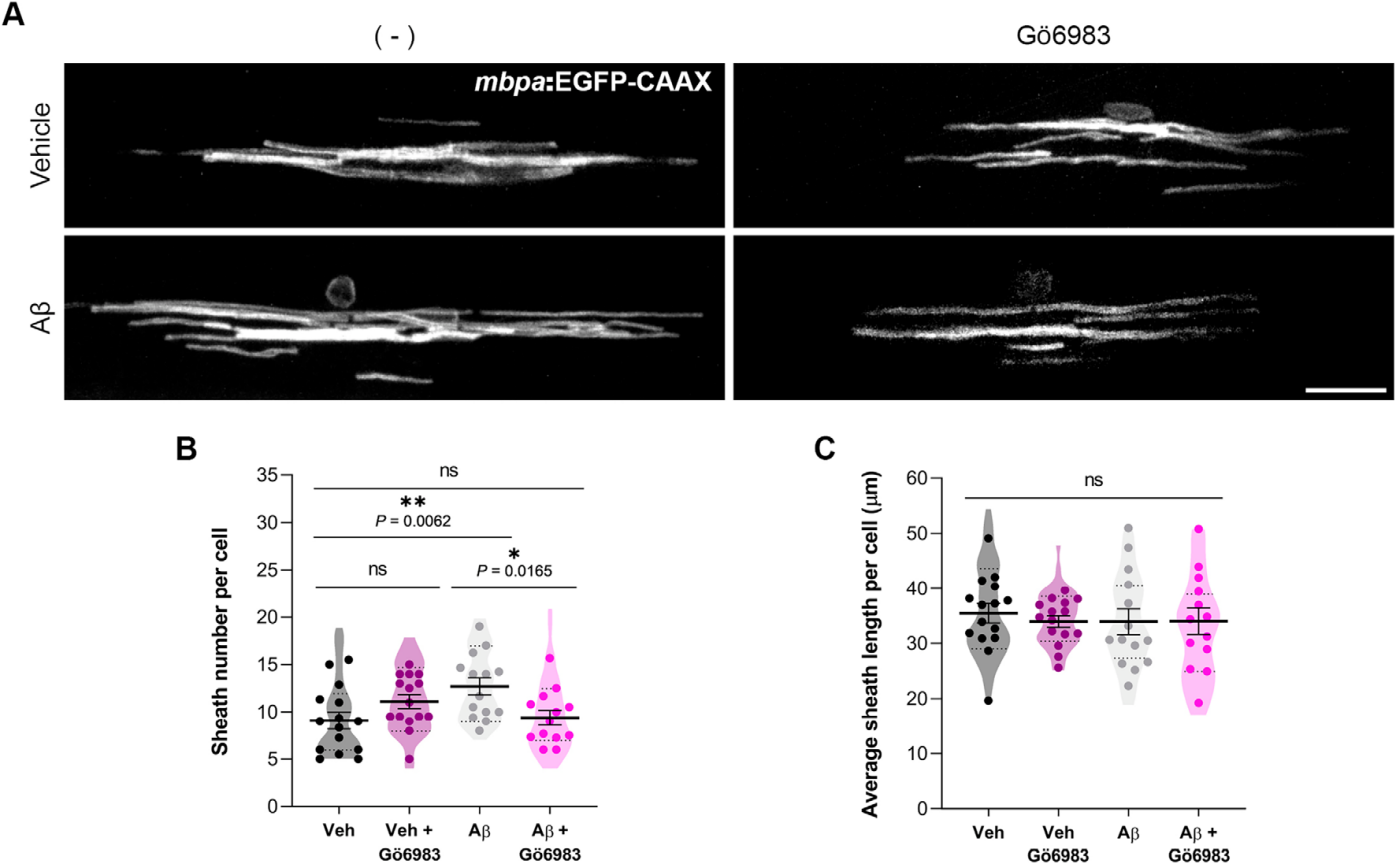
Aβ dysregulates dorsal myelin sheath number per oligodendrocyte via PKC activation. The *mbpa*:EGFP‐CAAX plasmid was injected into zebrafish embryos at the 1‐cell stage, followed by the administration of 10 μM of Aβ or its vehicle into the hindbrain ventricle of zebrafish larvae at 24 hpf. 500 nM Gö6983 treatments were performed at 48 hpf, and live imaging of myelin sheaths was performed at 5 dpf. (A) Representative fluorescent images of OLs in the spinal cord of live zebrafish larvae. (B) Histograms illustrating the sheath number per cell and (C) the average individual sheath length. Scale bar = 20 μm. Data are presented as means ± SEM; dots represent individual larvae and violin plots quantification of individual cells. *p < 0.05, **p < 0.01; Statistical significance was determined by two‐way ANOVA followed by Sidak's *post hoc*. *n* = 13–15 larvae per condition.

Aβ significantly increased the number of myelin sheaths per cell by around 40% in the dorsal spinal cord compared to vehicle‐injected animals. Interestingly, while PKC inhibition alone did not significantly alter sheath numbers, treatment of Aβ‐injected zebrafish larvae with Gö6983 restored the aforementioned Aβ‐induced increase to control levels (vehicle 9.08 ± 0.9 sheaths; Gö6987 11.1 ± 0.73 sheaths; Aβ 12.7 ± 0.9 sheaths; Aβ + Gö6983 9.38 ± 0.79 sheaths) (Figure 5A,B). This change in sheath number per cell occurred without any alteration in myelin sheath length (Figure 5C).

**FIGURE 6:**
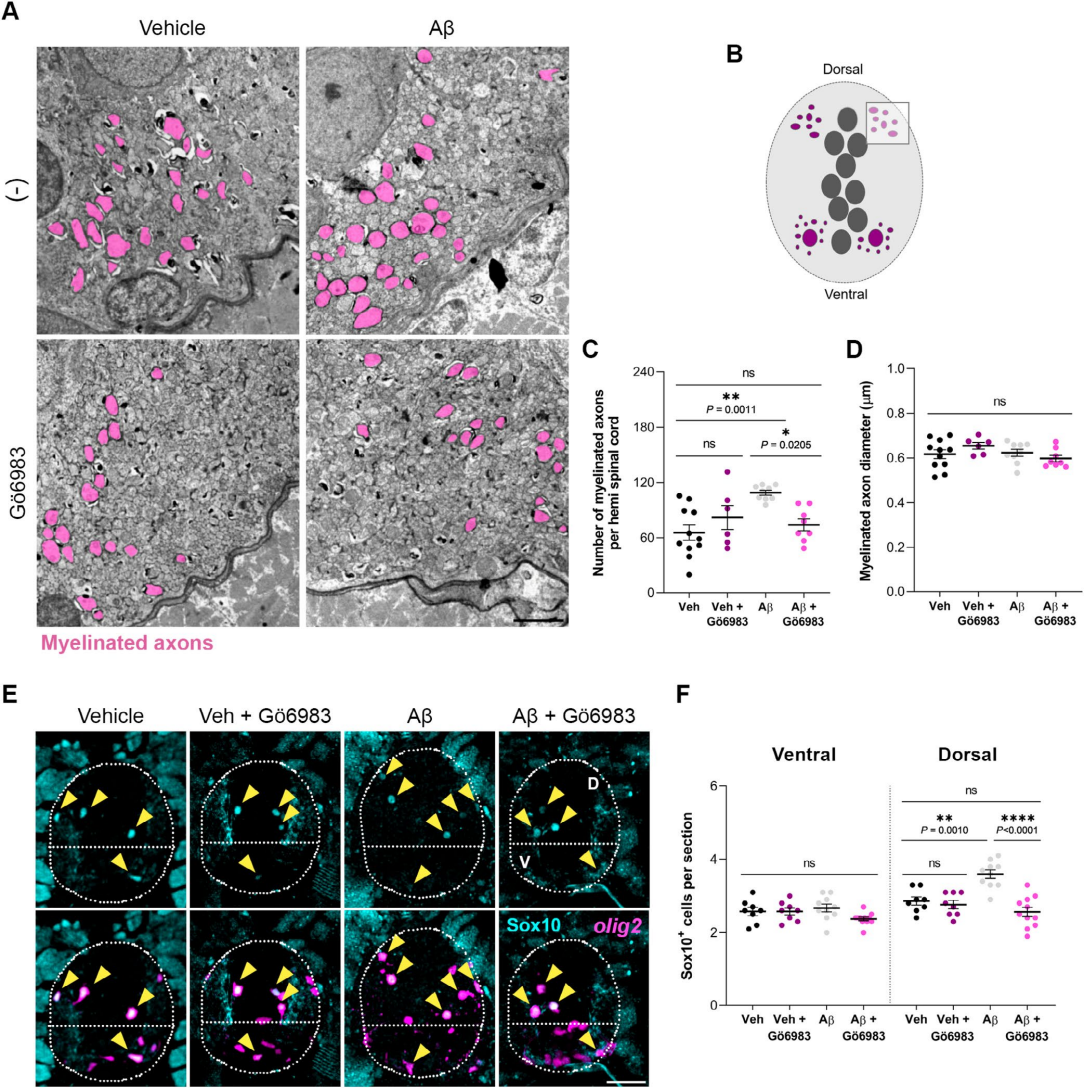
Aβ increases the number of dorsal oligodendroglial lineage cells and myelinated axons through PKC. Zebrafish larvae were intracerebroventricularly injected with Aβ (10 μM) or its vehicle at 24 hpf, and some were exposed to Gö6983 (500 nM) at 48 hpf. Larvae were fixed for electron microscopy or immunohistochemistry at 8 dpf. (A) Representative electron micrographs of the dorsal spinal cord of 8 dpf zebrafish larvae, with myelinated axons shaded in pink. (B) Schematic illustration of a cross‐section of the zebrafish spinal cord. The square represents the analyzed area. Histograms showing the (C) number and (D) diameter of myelinated axons in the dorsal area for each condition. Scale bar = 2 μm. *n* = 6–11 larvae per condition. (E) Representative transverse sections of *olig2*:EGFP (magenta) larvae spinal cords processed for immunohistochemistry to detect Sox10 expression (cyan; yellow arrowheads). Dashed white line indicates the outer edge of the spinal cord and dorsal and ventral areas. (F) Quantification of Sox10^+^ cells in the ventral and dorsal spinal cord at 8 dpf. Scale bar = 20 μm. *n* = 8—11larvae per condition. Data indicate means ± SEM, with dots representing individual larvae. *p < 0.05, **p < 0.01, ****p < 0.0001. Statistical significance was determined by two‐way ANOVA followed by Sidak's and Tukey's *post hoc* tests.

Next, we questioned whether the observed increase in the number of dorsal myelin sheaths per cell might lead to significant changes in overall myelination. To address this, electron microscopy was conducted on 8 dpf zebrafish larvae, and the myelinated axons within the dorsal region of the spinal cord were analyzed (Figure 6A,B).

In line with our previous findings, a significant increase of approximately 66% in the number of dorsal myelinated axons was observed in Aβ‐injected zebrafish larvae compared to vehicle‐injected larvae (108.9 ± 2.64 and 65.65 ± 8.24 myelinated axons, respectively). Moreover, while Gö6983 alone had a minimal nonsignificant impact on dorsal myelination (81.98 ± 12.96 myelinated axons), PKC inhibition in Aβ‐injected larvae nearly completely reversed the myelin excess induced by Aβ (74.07 ± 6.44 myelinated axons) (Figure 6C).

Finally, to assess potential changes in the number and distribution of oligodendrocyte lineage cells at 8 dpf, we performed immunohistochemistry to detect Sox10 in *Tg*(*olig2:EGFP*)^*vu12*^ larvae (Figure 6E). Quantification revealed that although the number of Sox10^+^ cells across groups was unchanged in the ventral spinal cord, it was significantly higher in the dorsal area of Aβ‐injected larvae compared to vehicle‐injected larvae, an effect that was reverted after PKC inhibition (vehicle 2.86 ± 0.11 cells; Gö6987 2.76 ± 0.11 cells; Aβ 3.59 ± 0.12 cells; Aβ + Gö6983 2.56 ± 0.13 sheaths) (Figure 2F).

Altogether, we demonstrated that Aβ alters oligodendroglial lineage cell dynamics, leading to increased myelination, and we identified PKC as a key player in Aβ‐induced dysregulation in vivo.

## 4 Discussion

Growing evidence suggests that OL and myelin damage play significant roles in AD, potentially leading to neuronal dysfunction and cognitive decline. However, the effects of Aβ on OL differentiation and myelination in vivo remain unclear. Zebrafish have emerged as a powerful model for studying oligodendroglial cells and myelin biology due to their optical transparency during early developmental stages, rapid external development, and genetic tractability. Notably, many genes and pathways associated with OL lineage biology and myelination are highly conserved across species. These features make zebrafish a valuable model for investigating mechanisms relevant to human health and disease (Choi et al. 2021; Masson and Nait‐Oumesmar 2023; Preston and Macklin 2015).

In this study, we investigated the effects of ICV injection of Aβ on oligodendroglial lineage cells and myelin in the developing spinal cord of zebrafish larvae. For that, we focused on the expression of two well‐established genes known to play pivotal roles in OL differentiation and myelination: *myrf*, primarily expressed in differentiating OLs, and *mbp*, expressed in mature myelinating OLs. Our analysis revealed that Aβ administration leads to alterations in the expression levels of both *myrf* and *mbp* mRNAs during larval development. Initially, *myrf* expression was significantly elevated in Aβ‐injected larvae but decreased by 5 dpf. Consistently, recent research has also reported elevated MYRF levels in early‐stage AD patients, followed by a significant decline in later stages (Gabitto et al. 2024). Consistent with *myrf*, the *mbp* expression showed a notable increase in the presence of Aβ at early developmental stages, which was then normalized to control levels as development progressed. This finding was surprising, considering that myelination typically commences around 72 hpf (Buckley et al. 2010) and *mbp* expression is usually barely detectable at 48 hpf, as confirmed by the vehicle‐injected zebrafish. The early induction of *mbp* mRNA observed in Aβ‐injected larvae at 48 hpf suggested an accelerated myelination process in the presence of Aβ. Furthermore, we also identified *mbp* transcript accumulation in the dorsal region of the spinal cord, indicating enhanced OL maturation and migration. Consistently, using live imaging of transgenic reporter lines for *olig2*, *myrf*, and *mbp* transcripts, we found that Aβ exposure increased the number of *myrf* ^+^ and *mbp* ^+^ OLs in the dorsal spinal cord at 72 hpf and 5 dpf, respectively, without altering the total *olig2* ^+^ cells. Unexpectedly, we observed a significant increase in the number of Sox10^+^ cells in the dorsal area at 8 dpf in Aβ‐treated larvae. Changes in the number and dynamics of oligodendroglial lineage cells are common features observed in both physiological aging and neurodegenerative disorders like AD. Our findings align with previous studies demonstrating Aβ‐induced OL differentiation and maturation in organotypic cerebellar slices and primary OL cultures (Quintela‐López et al. 2019). Similarly, increased oligodendrogenesis (Desai et al. 2010; Ferreira et al. 2020) and a higher number of oligodendroglial cells (Behrendt et al. 2013; Morrissey et al. 2024) have been observed during the early stages of AD in mouse models. However, postmortem analyses of AD patients' tissues have shown a decline in Olig2^+^ cells (Behrendt et al. 2013), and a reduction in OPC density has been observed in the APP/PS1 (Chacon‐De‐La‐Rocha et al. 2020) and 5xFAD model (Zota et al. 2024). In contrast, recent analyses of 12‐month‐old APP/PS1 mice reported unchanged oligodendrogenesis but a significant loss of OLs in the hippocampus (DeFlitch et al. 2022). These inconsistencies in oligodendrocyte responses to AD remain unexplained but may arise from differences in brain regions examined, experimental models employed, or disease stages analyzed. In our study, the observed precocious differentiation and the increase in oligodendroglial lineage cells at 8 dpf may result from enhanced differentiation, increased proliferation, and/or cell viability. Notably, soluble forms of Aβ have been shown to promote oligodendrocyte survival in vitro (Quintela‐López et al. 2019). However, further research is needed to fully elucidate the mechanisms behind this effect. Despite being based on a developmental model, our study aligns with findings in AD, suggesting potential parallels between Aβ‐induced oligodendroglial changes during development and neurodegeneration.

Interestingly, we also demonstrated that Aβ increases the number of myelin sheaths per OL in the dorsal spinal cord of zebrafish larvae, with no changes in sheath length. This result is consistent with findings from a previous study where a transgenic zebrafish line with constitutively active Fyn kinase in all myelinating OLs showed an increase in sheath number per cell, without alterations in OL number or myelin sheath length (Czopka et al. 2013), which is comparable to what we observed in Aβ‐injected zebrafish larvae. This suggests a potential involvement of Fyn kinase in mediating the effects of Aβ on myelination in vivo. Interestingly, Fyn has previously been described as a downstream target of Aβ both in vitro and in animal models (Boehm 2013; Quintela‐López et al. 2019). Furthermore, we observed elevated numbers of myelinated axons in the dorsal spinal cord of the zebrafish larvae exposed to Aβ. Interestingly, another study found that while overall myelination levels were reduced in the hippocampus of APP/PS1 mice, the formation rate of new myelin sheaths was higher compared to control mice (Chen et al. 2021).

However, increased myelination does not necessarily indicate a beneficial outcome. The precise and coordinated production of myelin is essential for the correct development and functioning of the CNS. For instance, in epilepsy, irregular neuronal activity has been shown to enhance oligodendrogenesis and myelination, resulting in maladaptive myelination that may contribute to disease progression (Knowles et al. 2022). In transgenic AD mouse models, multiple studies have reported myelin abnormalities (Behrendt et al. 2013; Desai et al. 2009; Desai et al. 2010). In addition, it was described that myelin plays a critical role in promoting the deposition of Aβ plaques and pointed out that maintaining myelin integrity might be a promising therapeutic strategy to delay or mitigate the disease (Depp et al. 2023). More interestingly, previous studies have shown that Aβ‐injected zebrafish exhibit reduced avoidance of aversive stimuli compared to animals injected with vehicle alone (Nery et al. 2014). Consequently, it is plausible to speculate that the hypermyelination caused by the precocious differentiation of OLs in our Aβ‐injected zebrafish larvae may be aberrant or dysfunctional in the long term, potentially affecting axonal functionality and viability; however, further investigations are needed to confirm this hypothesis. These results together highlight the relevance of maintaining appropriate transcription factor levels and OL differentiation timing during development.

Aβ are promiscuous molecules able to signal through a repertoire of receptors and signaling pathways, promoting a wide range of effects in neurons, but also in OLs (Viola and Klein 2015). Therefore, elucidating the underlying molecular mechanisms of Aβ is both of the utmost importance and challenging. In this study, we focused on PKC as a candidate kinase to modulate the Aβ‐induced signaling pathway leading to OL and myelin alterations. The diversity of PKC isoforms and the range of available inhibitors add complexity to this field of study, resulting in discrepancies in the literature. Some researchers report therapeutic effects of PKC activation in AD models (Etcheberrigaray et al. 2004), while others show that specific inhibition of PKCδ reverses AD phenotypes (Du et al. 2018). Nevertheless, the impact of PKC on OL and myelin dysfunctions has not been addressed. Here, we demonstrated that PKC inhibition with the pan‐PKC inhibitor Gö6983 effectively reverses Aβ‐induced precocious OL differentiation and maturation and oligodendroglial cell number increase, as well as the resulting hypermyelination. Importantly, this outcome not only confirms PKC's involvement in these biological processes and the Aβ‐induced signaling, but it also highlights Gö6983's efficacy in mitigating OL and myelin alterations induced by Aβ in the developing spinal cord of zebrafish larvae. Interestingly, regarding the previously mentioned role of Fyn kinase in myelin modulation (Czopka et al. 2013), our findings suggest that PKC inhibition counteracts the effects of Fyn kinase on myelin, indicating a potential common pathway for both kinases. However, whether PKC acts upstream or downstream of Fyn remains to be elucidated.

Therefore, as we demonstrated that soluble Aβ has an effect in oligodendroglial lineage cells and myelination, and considering recent research showing that OLs produce Aβ alongside neurons in AD mouse models (Rajani et al. 2024; Sasmita et al. 2024) and human brains (Gazestani et al. 2023), it is plausible to hypothesize that OL‐derived Aβ may play a physiological role in modulating myelination, possibly acting as a regulatory signal. Indeed, mature OLs have been described as producing more Aβ compared to OPCs. This idea aligns with our previous findings in neurons, where Aβ oligomers were shown to influence neuronal structure, including early effects on dendritic spine dynamics and arborization in hippocampal neurons (Ortiz‐Sanz et al. 2020). Collectively, these findings suggest that Aβ signaling may be involved in broader physiological neurodevelopmental and neuroplasticity processes, including OL differentiation and myelination, which could become dysregulated under pathological conditions such as AD.

In summary, our study demonstrates that Aβ disrupts oligodendroglial lineage cell differentiation, dynamics, and myelination during zebrafish development through a PKC‐dependent pathway. While this research was conducted in a developmental model, the findings provide valuable insights into the mechanisms underlying OL and myelin dysfunction in the context of amyloidosis, and potentially AD, where maladaptive myelination may contribute to disease progression. The conservation of OL biology between zebrafish and mammals supports the relevance of these findings and highlights PKC as a potential therapeutic target. Although further research is needed to explore these pathways and their roles in AD progression, this study underscores the importance of maintaining proper OL dynamics and myelination for both healthy development and potential disease mitigation.

## Author Contributions

U.B. and A.G.‐B. designed and performed the experiments, analyzed and interpreted data, and wrote the manuscript. C.A.K., L.B.‐C. and M.V.S.‐G. performed the experiments and analyzed and interpreted data. J.L.Z. provided help to design the project, with data interpretation, and reviewed the manuscript. E.A. and B.A. contributed to the conception and design of the project, analyzed and interpreted data, and wrote the paper. All authors have read and approved the final manuscript.

## Ethics Statement

All zebrafish work was approved by the Institutional Animal Care and Use Committee at the University of Colorado School of Medicine.

## Conflicts of Interest

The authors declare no conflicts of interest.

## Supporting information


**Data S1.** Supporting Information.

**Figure S1.** (A) Intraventricular injection of fluorescently labeled dextran into 24 hpf zebrafish larvae. (B) Western blot of Aβ species in the injection mixture; monomers and different types of oligomers.

**Figure S2.** Aβ activates PKC in primary cultured oligodendrocytes. (A) Western blot analysis and (B) quantification of PKC phosphorylation levels in control and Aβ‐treated OLs. Histogram represents protein expression levels as percentages (%) relative to control cells. Data are presented as means ± SEM; dots represent individual experiments and violin plot represents quantification of individual cells. **p < 0.01; Statistical significance was determined by one‐way ANOVA followed by Dunnett’s *post hoc* test.

**Figure S3.** *Myrf* ^+^ cell numbers are unchanged at 5 dpf. (A) Representative lateral images of the spinal cord of live transgenic larvae stably expressing *olig2*:EGFP and *myrf*:mScarlet, at 5 dpf. Graphs showing the number of (B) total and (C) dorsal *myrf* ^+^ cells. (D) Pie charts showing the ratio of differentiating dorsal OLs (percentage of dorsal *myrf* ^+^ cells among dorsal *olig2* ^+^ cells) at 5 dpf for each condition. Scale bar = 20 μm. Data indicate means ± SEM, and dots represent individual larvae (*n* = 7–9 larvae per condition and time point). Statistical significance was determined by two‑way ANOVA followed by Sidak’s *post hoc* test.

**Figure S4.** *Mbp* ^+^ cell numbers are unchanged at 72 hpf. Graphs showing the number of (A) total and (B) dorsal *mbp* ^+^ cells in the spinal cord of live transgenic larvae stably expressing *olig2*:EGFP and *mbpa*:tagRFPT, at 72 hpf. (C) Pie charts showing the ratio of mature myelinating dorsal OLs (percentage of dorsal *mbp* ^+^ cells among dorsal *olig2* ^+^ cells) at 72 hpf for each condition. Data indicate means ± SEM, and dots represent individual larvae (*n* = 6–12 larvae per condition and time point). Statistical significance was determined by two‑way ANOVA followed by Sidak’s *post hoc* test.

## Data Availability

The data that support the findings of this study are available from the corresponding author upon reasonable request.
