# Supplementary material for: Amyloid‐β Dysregulates Oligodendroglial Lineage Cell Dynamics and Myelination via PKC in the Zebrafish Spinal Cord
Source: Glia. 2025 Mar 14;73(7):1437–51. doi: 10.1002/glia.70015 (PMC12121469; doi:10.1002/glia.70015)
Supplement: Supplementary file 1 — Data S1. Supporting Information. [file GLIA-73-1437-s001.pdf]

Amyloid β dysregulates oligodendroglial lineage cell dynamics and myelination via PKC in the zebrafish spinal cord

SUPPLEMENTARY METHODS

Intracerebroventricular injection of dextran in zebrafish larvae

At 24 hpf, all zebrafish embryos had their chorions removed for the brain ventricle injection procedure following previously established protocols (Gutzman & Sive, 2009; Nery et al., 2014). Embryos were anesthetized with Tricaine (Sigma-Aldrich) and microinjected in the hindbrain ventricle with 5–10 nl of a fresh injection buffer containing 10% fluorescein-labeled dextran (Invitrogen) and 10% Phenol Red in 0.4 M KCl in nuclease-free water. Subsequently, injected zebrafish were fixed in 4% PFA in PBS, gently rocking O/N at 4 ºC, and dextran spreading was visualized both in the brain and the spinal cord using a Zeiss Cell Observer 2D 25 Spinning Disk confocal system (Carl Zeiss Microscopy) with a 40X oil-immersion objective.

Cortical oligodendrocyte culture

Highly enriched OPCs were prepared from mixed glial cultures obtained from newborn (P_0–P_2) Sprague–Dawley rat forebrain cortices as previously described (McCarthy & De Vellis, 1980) with minor modifications (Sánchez-Gómez et al., 2018). Briefly, forebrains were removed from the skulls and the cortices were enzymatically and mechanically digested. Then, tissue was plated in Iscove's Modified Dulbecco's Medium supplemented with 10% fetal bovine serum. The mixed glial cells were grown in poly-D-lysine (PDL) treated T75 flasks until they were confluent (8–10 days). Microglia were separated from the cultures by shaking the flasks on a rotary shaker. OPCs were isolated by additional shaking for 18 h (200 rpm, 37°C, 5% CO2). OPCs were seeded on to PDL-coated coverslips at densities of $8x10^4$ cells/well and were maintained at 37 °C and 5% CO2 for 3 days in a chemically defined differentiation SATO medium.

Western blot

OLs were exposed to Aβ peptides as indicated. Cells were scraped in sodium dodecyl sulfate (SDS) sample buffer on ice to enhance the lysis process and avoid protein degradation. Samples were boiled at 95 °C for 8 min, size-separated in 4-20%

polyacrylamide-SDS Criterion TGX Precast gels (Bio-Rad) and transferred to Trans-Blot Turbo Midi Nitrocellulose Transfer Packs (Bio-Rad). Membranes were blocked in 5% BSA in Tris-buffered saline/0.05% Tween-20 and proteins were detected by specific primary antibodies against PKC (#ab179521, 1:1000; Abcam) and p-PKC (#9371, 1:1000; Cell Signaling). Membranes were incubated with horseradish peroxidase-conjugated secondary antibodies (1:5000). The protein bands were detected with a ChemiDoc XRS Imaging System (Bio-Rad), and quantified by using Image Lab 6.0.1 (Bio-Rad) software.

SUPPLEMENTARY FIGURES

Supplementary figure 1. (A) Intraventricular injection of fluorescently-labeled dextran into 24 hpf zebrafish larvae. (B) Western blot of Aβ species in the injection mixture; monomers and different types of oligomers.

Supplementary figure 2. Aβ activate PKC in primary cultured oligodendrocytes. (A) Western blot analysis and (B) quantification of PKC phosphorylation levels in control and Aβ-treated OLs. Histogram represent protein expression levels as percentages (%) relative to control cells. Data are presented as means ± S.E.M; dots represent individual experiments and violin plot represents quantification of individual cells. $^{**}p<0.01$; Statistical significance was determined by one-way ANOVA followed by Dunnett's *post-hoc* test.

Supplementary figure 3. *Myrf*$^{+}$ cell numbers are unchanged at 5 dpf. (A) Representative lateral images of the spinal cord of live transgenic larvae stably expressing *olig2*:EGFP and *myrf*:mScarlet, at 5 dpf. Graphs showing the number of (B) total and (C) dorsal *myrf*$^{+}$ cells. (D) Pie charts showing the ratio of differentiating dorsal OLs (percentage of dorsal *myrf*$^{+}$ cells among dorsal *olig2*$^{+}$ cells) at 5 dpf for each condition. Scale bar = 20 µm. Data indicate means ± S.E.M, and dots represent individual larvae (n = 7 – 9 larvae per condition and time-point). Statistical significance was determined by two-way ANOVA followed by Sidak's *post-hoc* test.

Supplementary figure 4. *Mbp*$^{+}$ cell numbers are unchanged at 72 hpf. Graphs showing the number of (A) total and (B) dorsal *mbp*$^{+}$ cells in the spinal cord of live transgenic larvae stably expressing *olig2*:EGFP and *mbpa*:tagRFPT, at 72 hpf. (C) Pie charts showing the ratio of mature myelinating dorsal OLs (percentage of dorsal *mbp*$^{+}$

cells among dorsal *olig2*$^{+}$ cells) at 72 hpf for each condition. Data indicate means ± S.E.M, and dots represent individual larvae (n = 6 – 12 larvae per condition and time-point). Statistical significance was determined by two-way ANOVA followed by Sidak's *post-hoc* test..
