# Supplementary figures and images for: Amyloid‐β Dysregulates Oligodendroglial Lineage Cell Dynamics and Myelination via PKC in the Zebrafish Spinal Cord
Source: Glia. 2025 Mar 14;73(7):1437–51. doi: 10.1002/glia.70015 (PMC12121469; doi:10.1002/glia.70015)

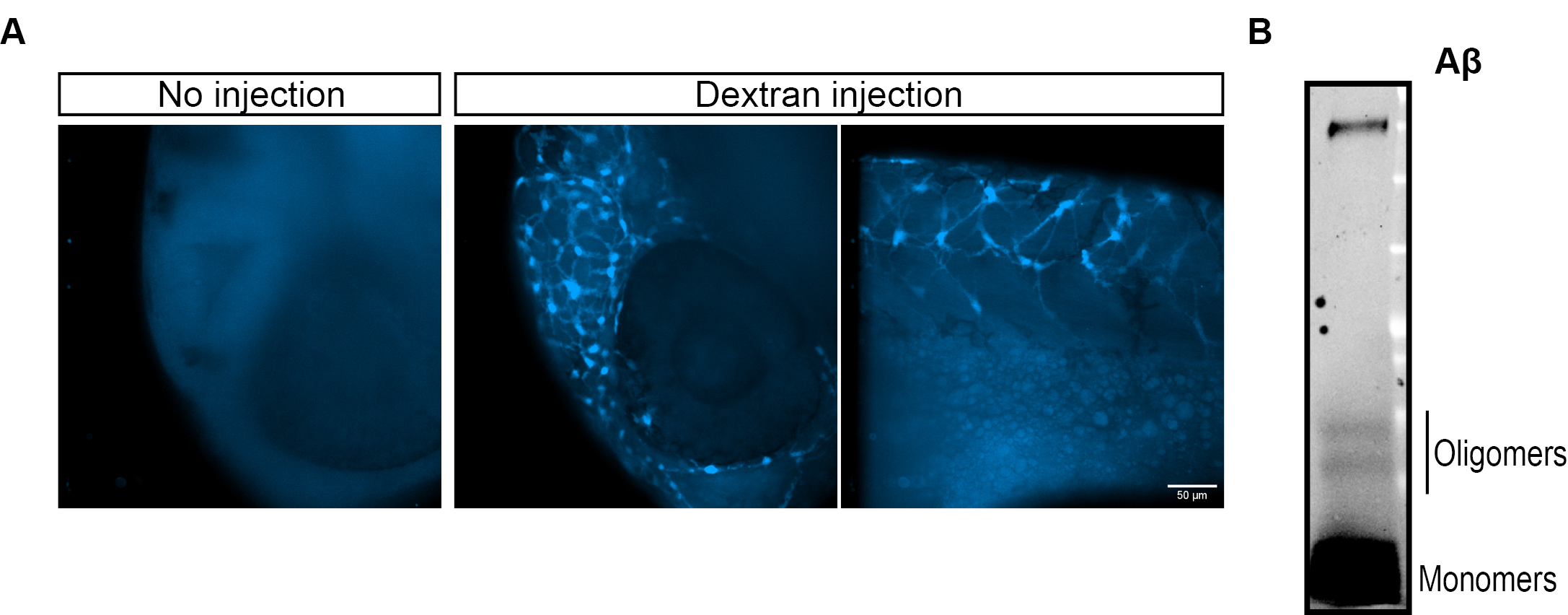

Supplement: Supplementary file 2 — Figure S1. (A) Intraventricular injection of fluorescently labeled dextran into 24 hpf zebrafish larvae. (B) Western blot of Aβ species in the injection mixture; monomers and different types of oligomers. [file GLIA-73-1437-s005.tif]

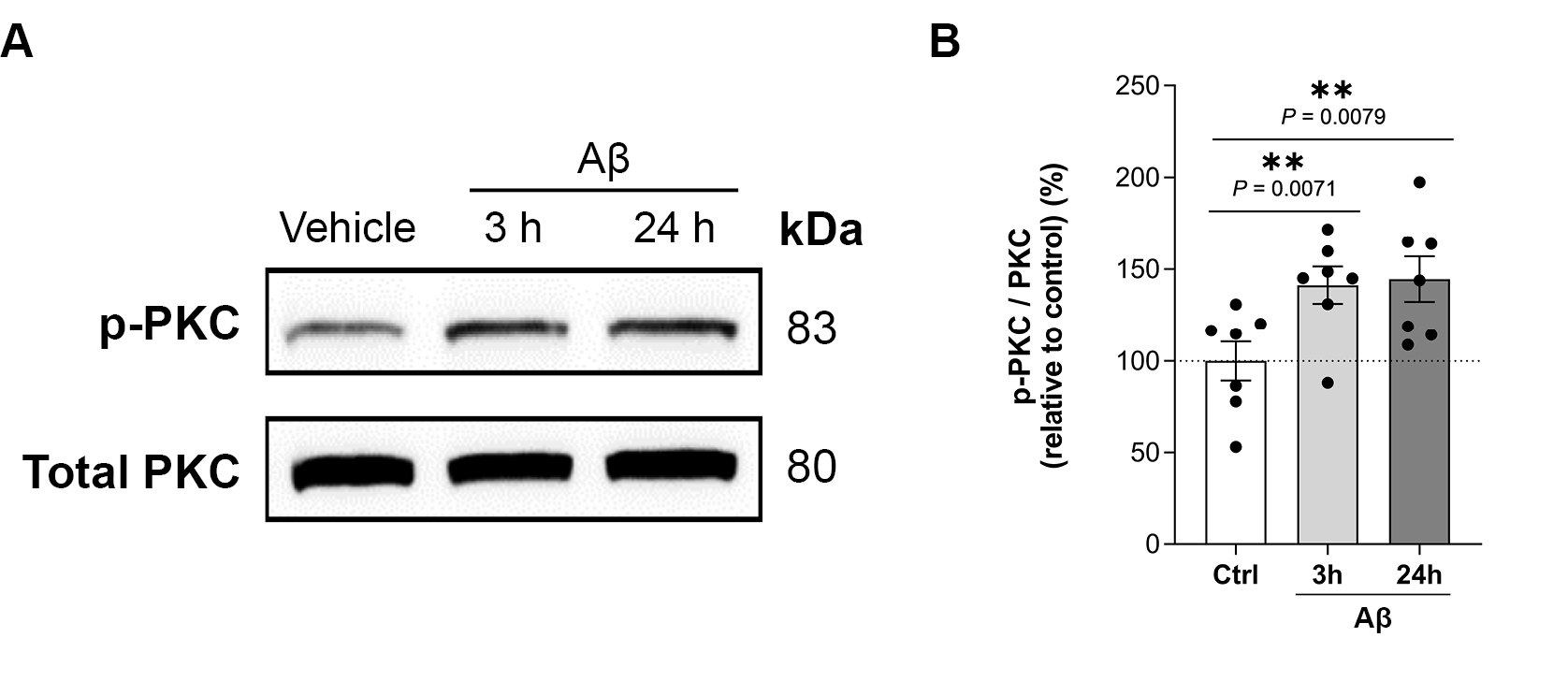

Supplement: Supplementary file 3 — Figure S2. Aβ activates PKC in primary cultured oligodendrocytes. (A) Western blot analysis and (B) quantification of PKC phosphorylation levels in control and Aβ‐treated OLs. Histogram represents protein expression levels as percentages (%) relative to control cells. Data are presented as means ± SEM; dots represent individual experiments and violin plot represents quantification of individual cells. **p < 0.01; Statistical significance was determined by one‐way ANOVA followed by Dunnett’s post hoc test. [file GLIA-73-1437-s003.tif]

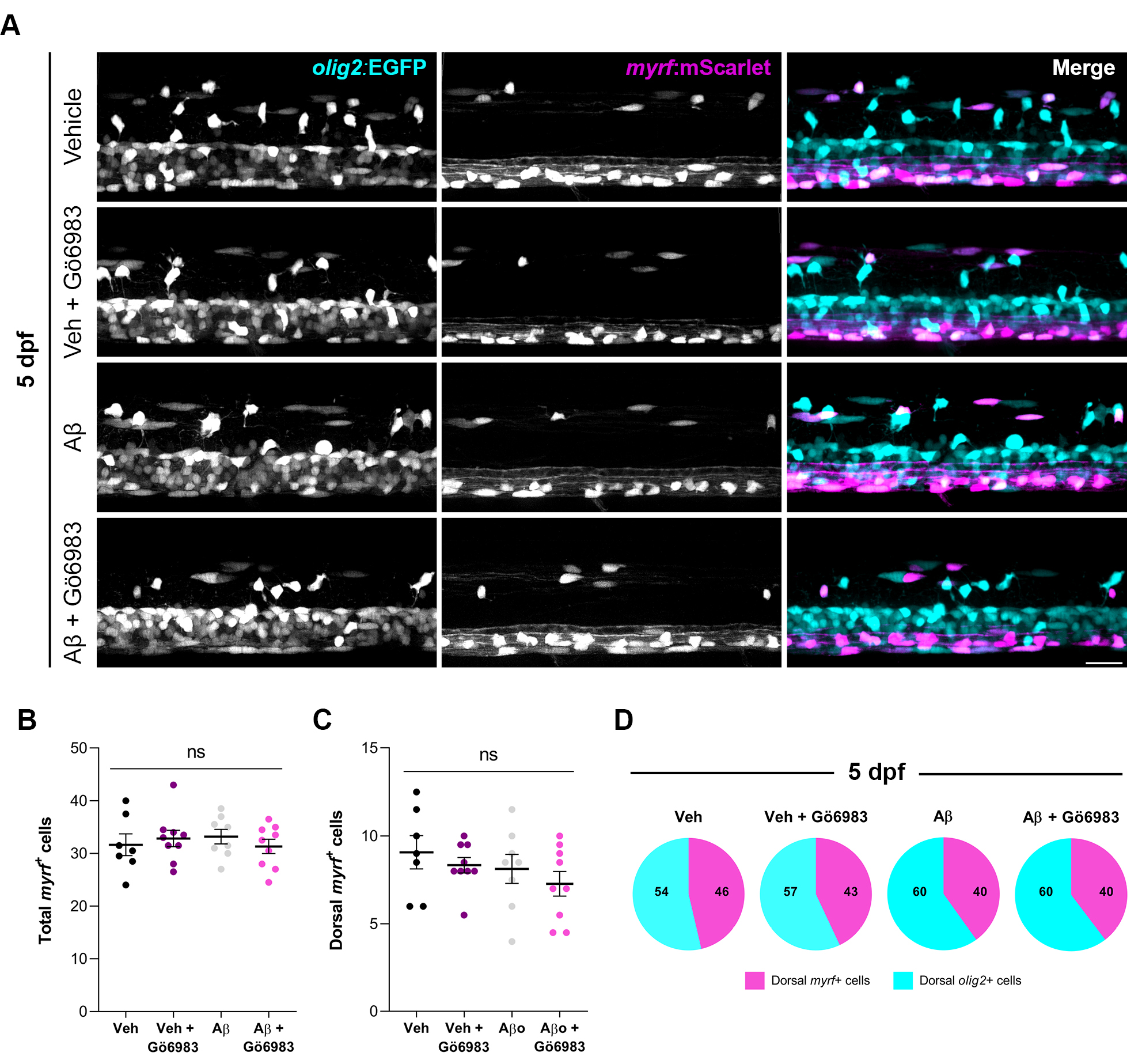

Supplement: Supplementary file 4 — Figure S3. Myrf + cell numbers are unchanged at 5 dpf. (A) Representative lateral images of the spinal cord of live transgenic larvae stably expressing olig2:EGFP and myrf:mScarlet, at 5 dpf. Graphs showing the number of (B) total and (C) dorsal myrf + cells. (D) Pie charts showing the ratio of differentiating dorsal OLs (percentage of dorsal myrf + cells among dorsal olig2 + cells) at 5 dpf for each condition. Scale bar = 20 μm. Data indicate means ± SEM, and dots represent individual larvae (n = 7–9 larvae per condition and time point). Statistical significance was determined by two‑way ANOVA followed by Sidak’s post hoc test. [file GLIA-73-1437-s002.tif]

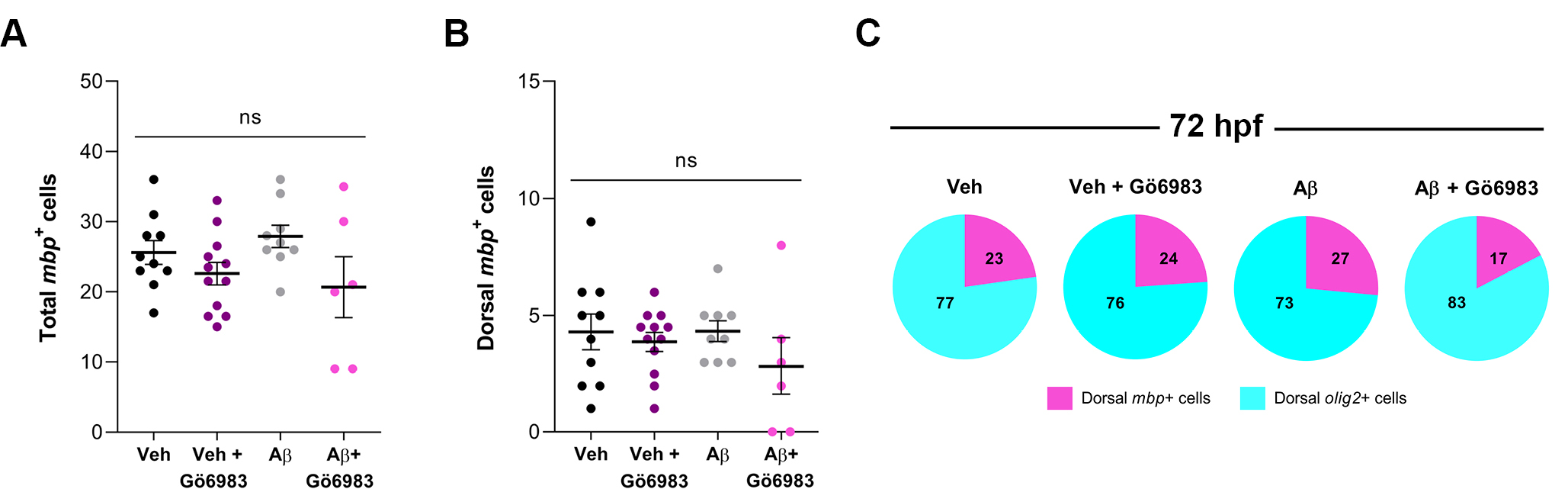

Supplement: Supplementary file 5 — Figure S4. Mbp + cell numbers are unchanged at 72 hpf. Graphs showing the number of (A) total and (B) dorsal mbp + cells in the spinal cord of live transgenic larvae stably expressing olig2:EGFP and mbpa:tagRFPT, at 72 hpf. (C) Pie charts showing the ratio of mature myelinating dorsal OLs (percentage of dorsal mbp + cells among dorsal olig2 + cells) at 72 hpf for each condition. Data indicate means ± SEM, and dots represent individual larvae (n = 6–12 larvae per condition and time point). Statistical significance was determined by two‑way ANOVA followed by Sidak’s post hoc test. [file GLIA-73-1437-s004.tif]
